# Energy Criterion for Fracture of Rocks and Rock-like Materials on the Descending Branch of the Load–Displacement Curve

**DOI:** 10.3390/ma15227907

**Published:** 2022-11-09

**Authors:** Gennady Kolesnikov, Vitali Shekov

**Affiliations:** 1 Institute of Forestry, Mining and Construction Sciences, Petrozavodsk State University, Lenin Pr., 33, 185910 Petrozavodsk, Russia; 2 Institute of Geology, Karelian Research Centre, Russian Academy of Sciences, Pushkinskaya St., 11, IG KarRC RAS, 185610 Petrozavodsk, Russia

**Keywords:** fracture mechanics, brittle materials, complete stress–strain curve, differential energy criterion fracture

## Abstract

This article deals with the problem of predicting the brittle fracture of rocks and similar materials, which can also include frozen sandy soils. Such materials, due to the diversity of their conditions of origin, are characterized by natural heterogeneity at the micro-, meso-, and macro-levels, which makes it difficult to develop sufficiently universal criteria for their strength. Despite a number of known models and criteria of strength and fracture, the search for such criteria remains an urgent problem. In this paper, using the energy approach to the mathematical modeling of mechanical systems, the fracture criterion is justified, which differs from the known criteria that do not require integration to calculate the strain energy $W_{e}$ and dissipation energy $W_{d}$. The well-known relation for the input energy $W=W_{e}+W_{d}$ is used. The object of the study was the ratio of $dW=dW_{e}+dW_{d}$. The main research question concerned what the ratio of $dW_{e}$ and $dW_{d}$ would be at the point of brittle failure. The search for an answer to the question led to the justification of a differential energy criterion for the failure of brittle materials on the descending branch of the full stress–strain curve. It was found that the point of predicted fracture is determined by the equality $\sigma =0.5\;\varepsilon E_{tangential}$ (if there is an inflection point on the ascending branch) or $\sigma =0.5\;\varepsilon E_{secant}$_secant. The main result of the work was ascertaining the differential strength and fracture criteria of brittle materials in the form of inequalities and equations, which were oriented for application in engineering calculations. Examples of application of the developed criteria are given; their consistency with the experimental data known from the literature confirmed.

## 1. Introduction

### 1.1. The Research Problem

Rocks, due to the diversity of their conditions of origin, are characterized by natural heterogeneity at the micro-, meso- and macro-levels. As a consequence, laboratory studies show that individual samples of the same rock type can exhibit somewhat different behavior and strength under the same influences [1,2,3]. These differences make it difficult to predict the behavior of rocks under natural and man-made influences (which are also inherently variable), so there is a set of topical problems, the solution of which is necessary to ensure sustainable development [4,5,6,7,8]. Studies aimed at solving the complexity of these problems are conducted in different directions. Accordingly, the problems can be classified according to different criteria, namely by the following: by separate directions; by research methodology within each direction; by the level of the problem (macro-, meso-, micro-level, according to the hierarchical nature of matter). Since the problem is multifaceted, methods of geophysics, geochronology, geochemistry, fracture mechanics and methods of mathematical modeling are used to predict the behavior of rocks [6,7,8,9,10]. Reviews of research in this area can be found in [1,2,3]. The direction of research in this paper was limited to models for predicting the fracture of rocks and similar materials. Numerous studies in this broad area use numerical and analytical modeling techniques, including artificial intelligence, machine learning, and artificial neural network algorithms [11,12,13,14,15]. These studies focus on analyzing brittle material behavior and predicting brittle fracture conditions [16,17]. What these studies have in common is that they use material failure criteria; certain strain, stress and strain energy ratios are usually used as such criteria, and other prediction criteria are also investigated in order to reduce the risk of brittle failure [18,19,20,21,22]. This paper is devoted to analytical modeling of the behavior of rocks and stone-like materials (e.g., concrete) under monotonically increasing loading and predicting the brittle fracture of the material taking into account the accumulated and dissipated strain energy.

### 1.2. Two Classes of Fracture Criteria for Brittle Materials

Numerous models of behavior and fracture of brittle materials can be systematized and classified according to various criteria. For our study, it was sufficient to consider two classes of models, taking into account only the scale of the object of study: micro- and meso-level models; macro-level models.

#### 1.2.1. Micro- and Meso-Level Models

Based on the fact that the presence of cracks is characteristic of brittle materials, a number of models of mechanical behavior and energy criteria have been developed, which are based on the analysis of the causes of damage near the crack tip. The current stage of development of these criteria is reflected, for example, in articles [23,24,25,26,27]. In the new approaches, in addition to the analysis of energy, strain and stress variations near the crack tip, the failure causes also include the influence of the fracture process zone (FPZ) [26]. For the theoretical justification of the criteria of the indicated class, methods of elasticity theory, and methods of mathematical modeling, are used. Laboratory test results are used to verify these criteria and the corresponding models [23,26]. Since the models of the mentioned class consider a small region of the material near the crack tip, these models can be referred to as micro- and meso-level models.

#### 1.2.2. Macro-Level Models

Another class of fracture criteria, and corresponding models of brittle material behavior, can include macro-level models and criteria. When justifying models and criteria of this class, it is explicitly or implicitly (by default) assumed that only part of the input energy is spent on deformation of the macro-object, while another part of the input energy is dissipated, both in the material and in the environment, and converted into other types of energy. Due to this, deformation of a real object is accompanied by gradual destruction and displacement of brittle material grains, friction over the grain–grain contact area, heating, acoustic emission and other physical effects [28,29,30,31,32,33]. Further description focuses on macro-level models, while crack evolution and other internal processes are not detailed, i.e., black-box methodology is used.

### 1.3. Working Hypothesis and Purpose of the Study

We used a frequently used approach, according to which a certain external force is required to fracture a material, which generates an input energy W, which can be divided into two parts [34,35]. One part of the energy ($W_{e}$) is spent on the elastic deformation of particles and bonds between particles; another part of the energy ($W_{d}$) is dissipated in the material and in the environment. Thus, at any moment of time, the following equation is fulfilled:

(1) $$W=W_{e}+W_{d},$$

There are no infinitely strong materials in nature, so a real material cannot accumulate and dissipate an infinite amount of energy. If the input energy W is too excessive for the given state of the material, then the above-mentioned bonds and/or material particles are destroyed and the excess energy is released. Depending on the loading conditions, there may be an explosive nature to the destruction, for example, of granite and basalt in uniaxial compression, which is reflected in the literature [36]. Thus, Equation (1) generally models the state of a brittle material, so it is of interest to compare the ratio of accumulated strain energy and dissipated energy for real materials, for example, for granite, sandstone, and basalt. Note that for ideal materials $\;W_{d}=0$.

Variants of such elastic-dissipative energy relations for brittle materials are known in the literature, but they are usually presented in integral form [37,38,39]. If integration is used, a load–load–displacement (or stress–strain) equation is necessary [38]. However, despite several important scientific results in this area, obtaining such equations is difficult because many different properties of real materials and loads must be considered [40,41,42]; e.g., the effect of test machine characteristics was studied in [36]. An analysis of the literature [43,44,45,46] showed that the differential criteria of strength remain understudied. To obtain such a criterion, using Equation (1) the relation (2) can be obtained:

(2) $$dW=dW_{e}+dW_{d}.$$

In physical terms, Equation (2) models the state of the specimen at time dt, when the material strain ε and stress σ change by dε and dε, respectively (or displacement u and load  F change by du and dF, respectively). To analyze the state of the material at any time interval dt using the differential fracture criterion, a complete load–displacement (or stress–strain) curve equation is needed, but integrating this equation to calculate energy is not required. 

Taking into account the above information, we formulated a working hypothesis: there is a certain relation $dW_{e}$ and $dW_{d}$ (Equation (2)), which can be used as an energy criterion of brittle materials failure in a differential form. Accordingly, we formulated the goal of the work: to construct a complete stress–strain curve, justify the energy criterion of brittle materials fracture in the differential form and perform verification of the developed criterion.

## 2. Methodology

### 2.1. Complete Stress–Strain Curve of a Brittle Material 

Brittle materials are characterized by micro- and meso-scale pores and cracks, whose development with increasing load leads to gradual destruction of a conglomerate of material particles [47], which is manifested in the non-linearity of the stress–strain diagram. The process of destruction of solids is ordered, and “the hierarchy of the scale of destruction begins with the size of the crystal lattice and continues up to the size of the tectonic plates in the geospheres” [48]. A review [49] showed that for a more complete understanding of the mechanical properties of heterogeneous materials it is necessary to consider that they are in some sense an intermediate link between the material and the structure. A model of such a structure can be a macro-object, consisting of meso-scale elements, the mechanical state of which, and their interaction with each other, determine the strength and stiffness of a brittle material [48,50].

**Figure 1:**
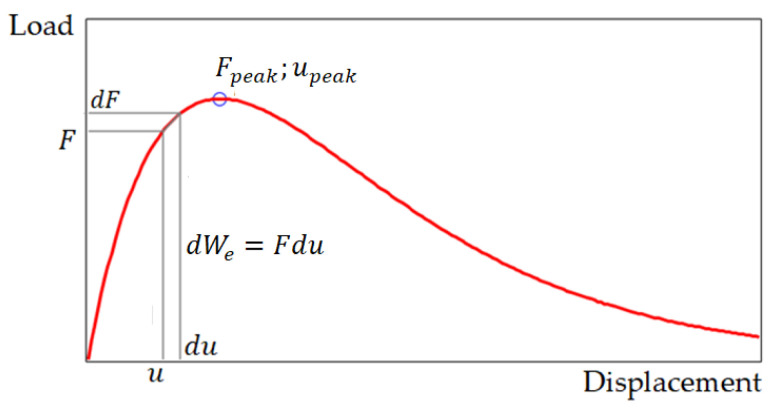
Load–displacement pattern.

A mathematical description of such a physical model is given in [51]; it was shown that the problem is reduced to the solution of Equation (3), which is known as the Furamura model (Figure 1) [52,53].

(3) $$F=F_{peak}\frac{u}{u_{peak}}e^{\left(1-\frac{u}{u_{peak}}\right)}$$

**Figure 2:**
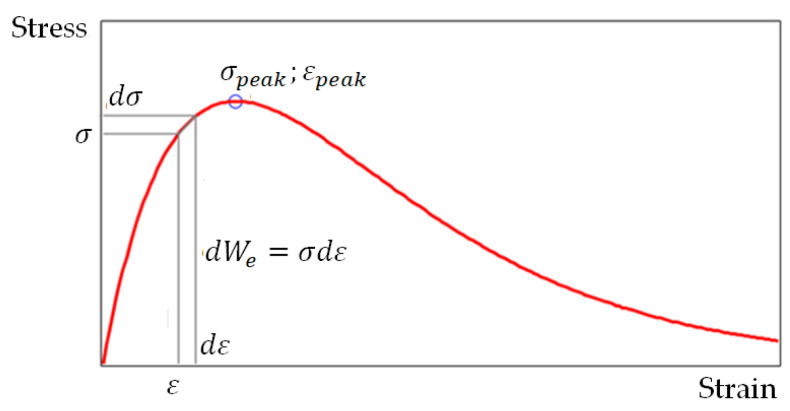
Stress–strain pattern.

Equation (3) models the relationship between the force and the displacement of its conditional point of application. In this work, it was necessary to switch to stress–strain terms in order to obtain comparative estimates of the destructive stresses and strains in compression of the brittle material samples, for example, in the form of a cylinder. To switch from load–displacement terms to stress–strain terms, we used the following relations: $\sigma =F/A_{0}$, $\sigma_{peak}=F_{peak}/A_{0}$, $\varepsilon =u/H_{0}$, $\varepsilon_{peak}=u_{peak}/H_{0}$; here $A_{0}$ and $H_{0}$ are cross-section area and sample height, respectively; it is assumed that all displacements are small. Using these relations, we transformed Equation (3) to the form (4) (see also Figure 2):

(4) $$\sigma =\sigma_{peak}\frac{\varepsilon }{\varepsilon_{peak}}e^{\left(1-\frac{\varepsilon }{\varepsilon_{peak}}\right)}$$

Equation (4) models the relationship between stress and strain in compression of a brittle material sample. It should be noted that not all brittle materials in compression show a pattern similar to Figure 2. In some cases, for example, when compressing granite and sandstone specimens [36], an inflection point appears on the ascending branch of the full stress–strain curve. Another feature of the full stress–strain curves for rigid brittle materials is that the descending branch of this curve is almost vertical if the material is in the stage of macro-crack growth with decreasing stress on the way to failure; at this stage, the axial stress decreases rapidly, accompanied by a small increase in deformation and an increase in the number of macro-cracks which coalesce and propagate through the sample volume. In uniaxial compression, explosive fracture can occur in, for example, granite, basalt and sandstone [36].

**Figure 3:**
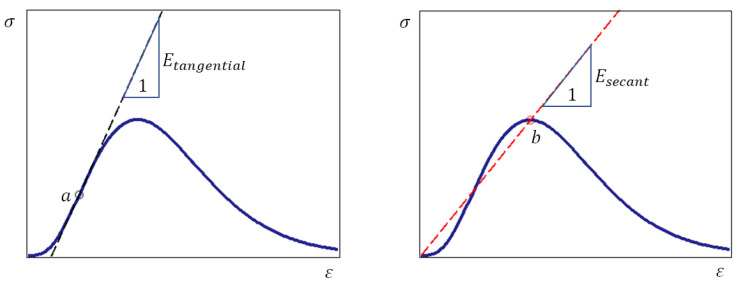
The line $\sigma =\varepsilon E_{tangential}$ passes through the inflection point a, the coordinates of which can be found from the equation $d^{2}\sigma /d\varepsilon^{2}=0$. The line $\sigma =\varepsilon E_{secant}$_secant passes through the origin and point b, where $\varepsilon =\varepsilon_{peak}$, $\sigma =\sigma_{peak}$ and dσ/dε=0.

Consider the stress–strain curve, which has the above-mentioned inflection point (Figure 3).

It is possible to draw infinitely many tangents to the curve in question. The angle of inclination of each of these tangents can be considered as the tangential modulus of elasticity. However, only point a on the ascending branch corresponds to the state in which the tangential modulus of elasticity E=dσ/dε reaches the highest values, because the condition of extremum of the function E=E(ε) is satisfied at this point: E=E(ε). From the physical point of view, the maximum value of the tangential modulus of elasticity is explained by the closure of cracks at a certain value of load, i.e., the real material at this stage of deformation is transformed into an almost ideal linearly elastic material. Therefore, if we compare a real brittle material at the model level with an imaginary ideal material without cracks, the tangential modulus of elasticity of the real material should be chosen as the modulus of elasticity of the ideal material (Figure 3).

However, mathematical description of complete curves with an inflection point on the ascending branch requires modification of Equation (4), because in the presented form this equation models only a particular case when there is no inflection point on the ascending branch of the stress–strain curve (Figure 2). Therefore, more universal models of approximately the same level of complexity have been developed [51,52,53]. In this research area, the works, [52,53] have indicated a tendency towards independent control of the branches of the full stress–strain curve (or load–displacement). Following this trend, let us perform decomposition of the Blagojevich model [52,53]:

(5) $$\sigma =\sigma_{peak}{\left(\frac{\varepsilon }{\varepsilon_{peak}}e^{\left(1-\frac{\varepsilon }{\varepsilon_{peak}}\right)}\right)}^{c};\;c=a,\;\mathrm{if}\;0\leq \varepsilon \leq \varepsilon_{peak};\;c=b,\;\mathrm{if}\;\varepsilon \geq \varepsilon_{peak}.$$

Parameters a and b are determined at the stage of model fitting (5). To determine the values of $\varepsilon_{peak}$ and $\sigma_{peak}$ experimental data are required; these values can be determined by direct or indirect methods, by analogy with [51,52,53].

Commenting on Figure 3, we note that the secant modulus of elasticity is used in engineering calculations [54]; for example, if there is no inflection point on the ascending branch of the stress–strain curve (Figure 2). Therefore, the secant modulus of elasticity is used in one of the variants of the fracture criterion, which is considered below.

### 2.2. Justification of the Energy Differential Fracture Criterion for Brittle Materials

Taking into account the working hypothesis formulated in Section 1.3, we assume that a compression test is performed on an ideal material whose tangential modulus is defined as shown above (Figure 3). In this case, the stress–strain relation is modeled by the linear equation $\sigma =\varepsilon E_{secant}$.

**Figure 4:**
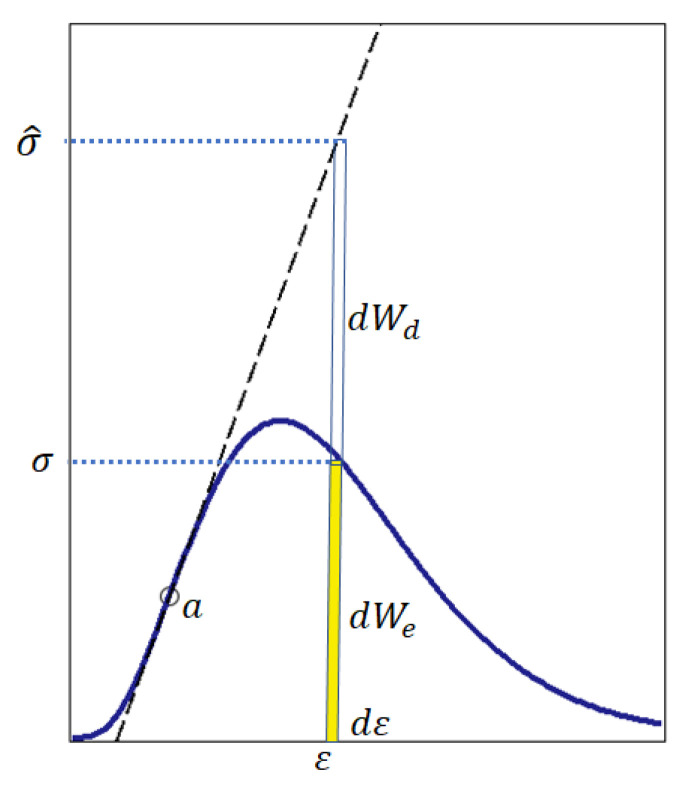
Strain energy $dW_{e}=\sigma d\varepsilon$ and dissipation energy $dW_{d}=\left(\hat{\sigma }-\sigma \right)d\varepsilon$. The stress in an ideal material without dissipation is $\hat{\sigma }=\varepsilon E_{tangential}$. The voltage σ in a material with energy dissipation is determined from Equation (5).

During loading, the weakest particles of the brittle material and particle–particle junctions collapse gradually, so that the load is redistributed over the not yet collapsed particles; therefore, the stress in these particles increases, but the number of undestroyed particles decreases, and in the post-peak state the bearing capacity of the sample decreases. Since weak particles are the first to collapse when the load increases, and the modulus of elasticity and strength correlate positively, it is reasonable to assume that the modulus of elasticity of the material of the undestroyed particles tend to increase. In contrast to an ideal material, in a real brittle material only part of the input energy is stored in the form of potential strain energy, the other part of the input energy is dissipated, which is modeled by Equation (3) and shown in Figure 4.

Equation (2) and Figure 4 induce two important questions. 

Question 1: If ε>0, is equality $dW_{d}=0$ possible? Answer: Yes, it is possible. Equality $dW_{d}=0$ is realized, for example, at point a (Figure 4).

Question 2: If ε>0, is equality $dW_{e}=0$ possible? Answer: If *ε* > 0, the equality $dW_{e}=0$ is impossible. In this case, according to Equation (2), $dW=dW_{e}+dW_{d}=0+dW_{d}=dW_{d}$. From a physical point of view, the equality $dW=dW_{d}$ means that the input energy is completely dissipated and the voltage σ=0, i.e., the material is non-functional. Consequently, if σ=0, then $dW_{e}>0$.

From the answers to questions 1 and 2 it follows: if ε>0, then that, for real brittle materials, the inequality is fulfilled:

(6) $$dW_{e}>dW_{d}.$$

From Equation (2) follows:

(7) $$dW_{d}=dW-dW_{e}.$$

Substitute $dW_{d}$ (7) into inequality (6):

(8) $$dW_{e}>dW-dW_{e}.$$

Inequality (8) is equivalent to inequality (9):

(9) $$dW_{e}>\frac{dW}{2}$$

From the physical point of view, inequality (9) means that the material is functional, i.e., the material resists the load if at any time the strain energy $dW_{e}=\sigma d\varepsilon$ is greater than half of the input energy $dW=\hat{\sigma }d\varepsilon =\varepsilon E_{tangential}d\varepsilon$. On this basis, taking into account the notations used above, the differential energy criterion of strength can be written in the form of inequality (10):

(10) $$\sigma d\varepsilon >\frac{1\;}{2}\;\varepsilon E_{tangential}d\varepsilon \;\mathrm{or}\;\sigma >\frac{1\;}{2}\;\varepsilon E_{tangential}.$$

Accordingly, the differential energy criterion for fracture of brittle material can be written in the form of inequality (11):

(11) $$\sigma \leq \frac{1\;}{2}\;\varepsilon E_{tangential}.$$

**Figure 5:**
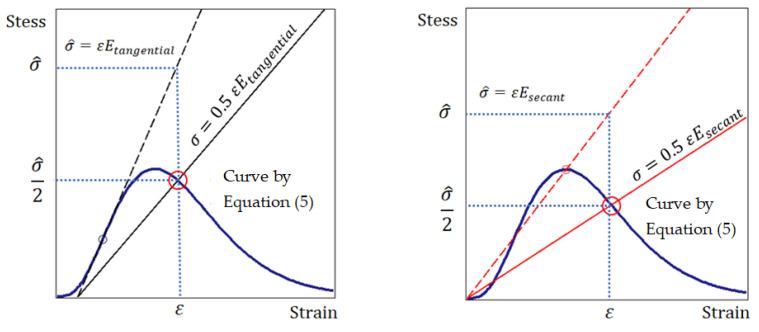
Fracture point on curve (5) (big red circle): using tangent (**left**) and secant modulus of elasticity (**right**).

The fracture point on the stress–strain curve is determined by equality (12) (Figure 5).

(12) $$\sigma =\frac{1\;}{2}\;\varepsilon E_{tangential}.$$

As noted above, in engineering calculations of the strength of brittle materials, e.g., concrete, the secant modulus of elasticity is used [54]. From the point of view of methodology, there are no fundamental differences in the justification of fracture criterion (11) using the secant modulus of elasticity instead of the tangential modulus of elasticity. Therefore, using the secant modulus of elasticity, by analogy with the criterion in the form of (11), we can write the fracture criterion in the form of (13); then, the predicted fracture point is determined by equality (14) (Figure 5).

(13) $$\sigma \leq \frac{1\;}{2}\;\varepsilon E_{secant}.$$

(14) $$\sigma =\frac{1\;}{2}\;\varepsilon E_{secant}.$$

The seeming illogic (sign≤) in fracture criteria (11) and (12) is explained by the fact that the stress σ is determined at a point on the descending branch of the full stress–strain curve Figure 2), that is, in this case the strain increases, but the stress decreases [50,55]. 

Figure 5 shows that failure occurs at the point where curve (5) crosses the line $\sigma =0.5\varepsilon E_{tangential}$ or $\sigma =0.5\varepsilon E_{secant}$. The stress σ is determined by Equation (5) depending on the strain ε; the tangential modulus of elasticity $E_{tangential}$ is determined taking into account the remarks in Figure 3. Thus, the use of the tangential and secant modulus of elasticity provides an interval of possible values of the fracture criterion. However, if there is no inflection point on the ascending branch of the stress–strain curve (Figure 2), only the secant modulus of elasticity is used.

## 3. Examples and Comparison with Experiments Known in the Literature

### 3.1. Example 1. Sandstone

**Figure 6:**
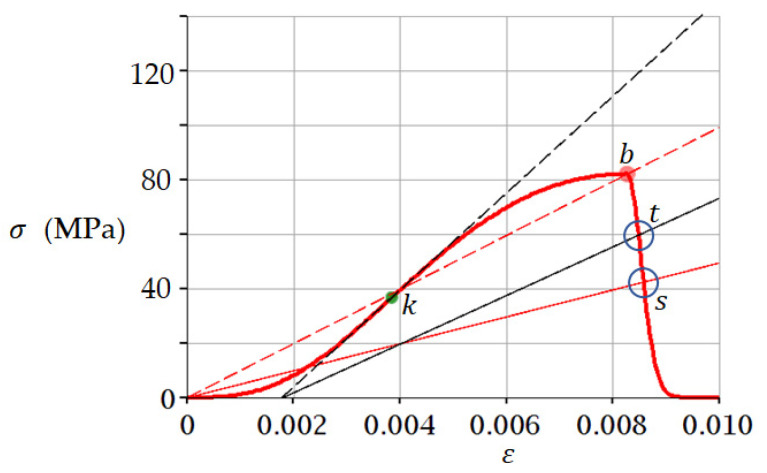
The stress–strain curve (5) for sandstone in uniaxial compression. A tangent (black dashed line) passes through point k, the slope angle of which determines the tangential modulus of elasticity. At point t, we predict failure according to criterion (12), at this point the line $\sigma =0.5\;\varepsilon E_{tangential}$ intersects the curve (5) (see also Figure 5). A secant (red dotted line) passes through point b, the slope angle of which determines the secant modulus of elasticity. At point s the failure is predicted by criterion (14), at this point the line $\sigma =0.5\;\varepsilon E_{secant}$ intersects the curve (5). The red curve simulates the experimental curve from [36]. The thin red and black lines correspond to Figure 5.

Let us consider an example of applying Equation (5) and the fracture criterion in the form of (12) and (14) to the analysis of sandstone compressive test results. The analysis is performed using the experimental data known from the literature [36], according to which $\sigma_{peak}=$ 82 MPa and $\varepsilon_{peak}=$ 0.00827 for sandstone. These data were substituted into Equation (5); values of parameters a and b were obtained by fitting: a= 3.5 and b= 1000. The stress–strain curve thus obtained is shown in Figure 6.

The coordinates of point k (Figure 6) are determined from the condition $d^{2}F/d\varepsilon^{2}=0$ using Equation (5): ε= 0.00385; σ= 36.64 MPa. According to criterion (12), failure is predicted at point t, for which ε= 0.00850; σ= 59.5 MPa. According to criterion (14), the destruction at point s, for which ε= 0.0086; σ= 42.5 MPa is predicted. In work [36] experimental values at a point of failure of sandstone at uniaxial compression were received: σ= 58.5 MPa; ε= 0.0091; stress at the fracture point almost coincides with the prediction by criterion (12): 58.5  ≈ 59.5 MPa. 

### 3.2. Example 2. Medium Coarse Sand (−10 °C)

**Figure 7:**
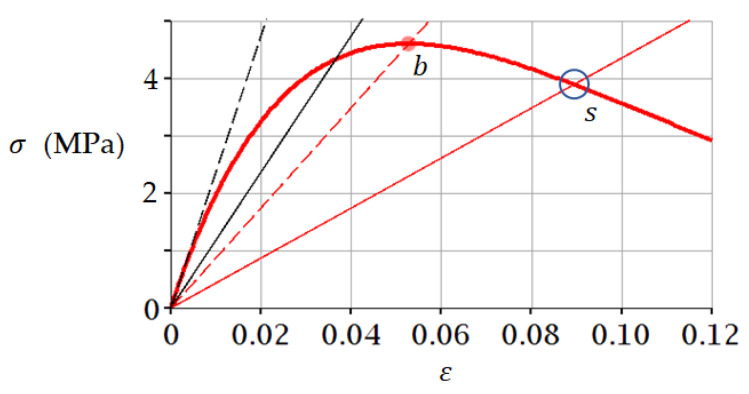
Stress–strain curve (5) for frozen sand under uniaxial compression. A secant (red dotted line) passes through point *b*, the slope angle of which determines the secant modulus of elasticity. At point s, failure is predicted by criterion (14), at this point the line $\sigma =0.5\;\varepsilon E_{secant}$ intersects the curve (5). The red curve simulates the experimental curve from [56].

Let us consider an example of application of Equation (5) and fracture criterion in the form of (12) and (14) to the analysis of test results of Medium coarse sand (−10 °C). The analysis is performed using the experimental data known in the literature [56], according to which $\sigma_{peak}=$ 4.6 MPa and $\varepsilon_{peak}=$ 0.0528. These data were substituted into Equation (5); values of parameters a and b were obtained by fitting: a= 1 and b= 1. The stress–strain curve thus constructed is shown in Figure 7.

In this case (Figure 7), there is no inflection point on the ascending branch of the stress–strain curve. Therefore, we will use criterion (14). According to criterion (14), the failure is predicted at point *s*, for which *ε* = 0.089; *σ* = 3.92 MPa. Predicted values almost coincide with the experimental data from work [56]: *ε* = 0.088; *σ* = 3.99 MPa. 

In the case under consideration, the tangent modulus of elasticity is determined by the angle of inclination of the black dotted line in Figure 7. In order to use the tangent modulus of elasticity to determine the failure point in accordance with criterion (12), it is necessary to construct a straight $\sigma =0.5\varepsilon E_{tangential}$ (solid black line in Figure 7) and justify the method of calculation, which, however, is beyond the scope of this paper. 

## 4. Discussion

The above examples show that the developed differential energy criterion for fracture of brittle materials (11)–(14) can be used to analyze brittle materials of both high and low stiffness. The examples discussed in Section 3 show that the parameter b for brittle materials (Example 1, b=1000) is much larger than that for a material of low stiffness (Example 2, b=1). Parameters a and b can be determined by the least-squares method, by analogy with papers [52,53], in which models of the same class are proposed. Parameters a and b were chosen according to test results. Analysis of the load–displacement curves showed that the values of parameters a and b correlated positively with the stiffness of the specimen [57]. We noticed that an empirical rule could be used: if there is an inflection point on the pre-peak branch of the load–displacement (or stress–strain) curve, parameter a can be determined from the condition of coincidence of the inflection points on the experimental and theoretical curve. The parameter b positively correlated with the absolute value of the post-peak modulus of elasticity, which can be used to determine the value of this parameter. The method of determination of post-peak elasticity modulus of granite, marble and other brittle materials is considered in article [58]. In addition, an empirical relationship linking the b parameter to the strain at the fracture point ($\varepsilon_{s}$) can be used: =(1−A/2)/(A−1); $A=\varepsilon_{s}/\varepsilon_{peak}$. This ratio is used if $\varepsilon_{s}>\varepsilon_{peak}$. These remarks indicate that parameters a, b,n in Equation (5) depend on the stiffness of the material [52,53,57], but the physical meaning of these parameters is not disclosed in this paper, which may be the subject of further research.

**Figure 8:**
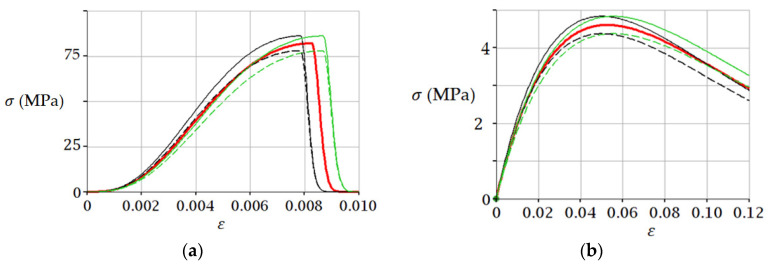
Effect of deviations in $\sigma_{peak}$, $\varepsilon_{peak}$ on uniaxial compression behavior of samples: (**a**) Sandstone from example 1; (**b**) Frozen sand from example 2. The red line corresponds to the parameters $\sigma_{peak}$, $\varepsilon_{peak}$. Thin lines correspond to parameters with deviations:$\sigma_{peak}\cdot \left(1\pm 0.05\right)\;$, $\varepsilon_{peak}\cdot \left(1\pm 0.05\right)$. The red curve simulates the experimental curve from [36] (**a**) and [56] (**b**).

The fracture point of brittle material under laboratory conditions depends on the characteristics of the testing machine, as shown in [36,58,59]. Hence, it follows that the parameters $\sigma_{peak}$, $\varepsilon_{peak}$ in Equation (5) can be determined with some deviations from true values. These deviations affect the simulation results. For example, if we assume, that parameters $\sigma_{peak}$, $\varepsilon_{peak}$ are defined with accuracy ±5%, then using Equation (5) we obtain for initial data from examples 1 and 2 the results shown in Figure 8.

Figure 8 shows that deviations of ±5% in values of parameters $\sigma_{peak}$ and $\varepsilon_{peak}$ do not lead to critical changes in predicted values of stresses using Equation (5), which indicates the possibility of the practical use of this equation. However, we should take into account the limitations of the presented model. Namely, Equation (3) models only the load–displacement relation, i.e., the external process. The development of cracks and other damages (internal process) is not directly considered. Thus, the presented model considers only input and output data without any specific knowledge of material properties, which corresponds to the “black box” methodology [60]. The input data are only the peak load and the corresponding displacement (peak displacement). This approach is justified in [51]; in this approach, the a priori damage variable is not used, but the hypothesis of stiffness (*dS*) and displacement (*du*) deterioration is introduced, which logically leads to Equations (3) and (4) [51].

The peak load and peak displacement are determined experimentally using direct or indirect measurement methods. When using the direct method, the test is carried out before failure, which is not always technically possible or economically feasible; in this case, the indirect measurement method is used. In [51,57], using frozen sandy soil as an example, it was shown that peak load and peak displacement could be determined (predicted) using experimental data for three points on the pre-peak branch of the load–displacement curve. In this case, there is no need to destroy the test object, but prediction errors appear, which decrease with increasing accuracy of experimental data, so it is necessary to use modern test machines [36,58], and appropriate instruments and equipment [59,60,61,62].

Other more versatile and accurate approaches are known for modeling the behavior of brittle materials under loading, and although such approaches require fairly accurate data on material properties, it is a relatively small price to pay for high accuracy in predicting brittle failure of engineering structures. The fracture process can be accurately investigated using fracture models, such as phase-field fracture [63,64,65]; a model of this class [63,64,65] is a complete model and is much more versatile than models (3), (4), (5) presented above. The advantage of models (3), (4), (5) is the small amount of initial data and the possibility to use them for prediction of the full load–strain curve from experimental data at three points on the pre-peak branch of the mentioned load–strain curve; besides, no prior knowledge of material properties is required, as shown by examples in [51,57].

## 5. Conclusions

This work uses the well-known load–displacement model for brittle material in uniaxial compression, which was justified in previous work using black-box methodology. The advantage of this methodology is that no specific knowledge of the physical and mechanical properties of the material is required in the simulation. The relationship between the strain energy $dW_{e}$ and the dissipation energy $dW_{d}$ was studied using this model.

It was proved that at the point of fracture on the post-peak stress–strain curve there is uniaxial compression $\;dW_{e}=dW_{d}$. Based on this relationship, two variants of the differential energy criterion for fracture on the post-peak curve stress–strain during uniaxial compression of a brittle material were obtained: $\sigma =0.5\;\varepsilon E_{secant}$, where $E_{secant}$ is the secant modulus of elasticity. If the pre-peak stress–strain curve has an inflection point, the fracture criterion has the form: $\sigma =0.5\;\varepsilon E_{tangential}$, where $E_{tangential}$ is the tangential modulus of elasticity.

The model and variants of the brittle material fracture criterion under uniaxial compression were verified using experimental data from the literature for frozen sandy soils.

## Data Availability

All data were calculated using the formulas given in the article. Experimental results known from the literature were used as input data for formula calculations. References to the corresponding publications are given in the text of the article.
